# Summary of the Standards, Options and Recommendations for the management of patients with nonmetastatic prostate cancer (2001)

**DOI:** 10.1038/sj.bjc.6601084

**Published:** 2003-08-15

**Authors:** A Villers, P Pommier, A Bataillard, B Fervers, J M Bachaud, N Berger, A F Bertrand, R Bouvier, D Brune, A Daver, E Fontaine, O Haillot, J L Lagrange, V Molinie, J P Muratet, P Pabot du Chatelard, M Peneau, D Prapotnich, V Ravery, P Richaud, D Rossi, M Soulie

**Affiliations:** 1 CHRU Hôpital Huriez, Lille, France; 2 Centre Léon Bérard, Lyon, France; 3 FNCLCC, Paris, France; 4 Institut Claudius Régaud, Toulouse, France; 5 Hôtel-Dieu, Lyon, France; 6 Centre Paul Papin, Angers, France; 7 Hôpital Edouard Herriot, Lyon, France; 8 Centre François Baclesse, Caen, France; 9 Hôpital Ambroise Paré, Boulogne, France; 10 CHU Bretonneau, Tours, France; 11 Hôpital Henri Mondor, Créteil, France; 12 Hôpital Foch, Suresnes, France; 13 Hôpital de la Source, Orleans, France; 14 Institut Mutualiste Montsouris, Paris, France; 15 CHU Bichat, Paris, France; 16 Institut Bergonié, Bordeaux, France; 17 CHU Hôpital Salvador, Marseille, France; 18 CHU Hôpital Rangueil, Toulouse, France

**Keywords:** nonmetastatic prostate neoplasm, practice guideline

In 1995 the crude incidence of prostate cancer in France was estimated to be 260–300 out of 100 000 for men aged between 60 and 70 years and over 500 out of 100 000 for men over 70 years of age. The majority of men (73%) are diagnosed when they are over 70 years old. The standardised incidence increased from 51.8 to 87.1 between 1985 and 1995. It has been estimated that in 1995, 26 474 new cases were diagnosed in France. The use of diagnostic tests has contributed to an increase in the number of patients detected with local or locoregional disease. The crude mortality rate for prostate cancer in France was 32.8 out of 100 000 in 1995, and remains stable.

## OBJECTIVES

The objectives of these recommendations are to define good clinical practice guidelines for the diagnosis and treatment of patients with nonmetastatic prostate cancer. The management of patients with stage T4 cancer is not specifically covered in this document, but some of the studies appraised also included patients with this stage of cancer.

## METHODS

The details of the methodology have been published previously (Fervers *et al*, 2001). For this particular SOR, a multidisciplinary group of experts was set up by the French National Federation of Cancer Centres (FNCLCC) and the French Urology Association (Association Française d'Urologie: AFU) to appraise critically the available evidence on the diagnosis and treatment of patients with prostate cancer. *Medline*® was searched from 1966 to 2000. This bibliography was completed by the references of the members of the working group, and a search for clinical practice guidelines published on the Internet. The working group selected and critically appraised pertinent references and then proposed the ‘Standards’, ‘Options’ and ‘Recommendations’ (SORs) for the diagnosis and treatment of patients with nonmetastatic prostate cancer, based on either the best available evidence or expert agreement.

‘*Standards*’ identify clinical situations for which there exist strong indications or contra-indications for a particular intervention and ‘*Options*’ identify situations for which there are several alternatives, none of which have shown clear superiority over the others (Table 1

**Table 1:** Definition of ‘Standards, Options and Recommendations’

Standards	Procedures or treatments that are considered to be of benefit, inappropriate or harmful by unanimous decision based on the best available evidence
	
Options	Procedures or treatments that are considered to be of benefit, inappropriate or harmful by a majority, based on the best available evidence
	
Recommendations	Additional information to enable the available options to be ranked using explicit criteria (e.g., survival toxicity) with an indication of the level of evidence

). In any SOR, there can be several ‘*Options*’ for a given clinical situation. ‘*Recommendations*’ enable the ‘*Options*’ to be weighted according to the available evidence. Several interventions can be recommended for the same clinical situation, so that clinicians can make a choice according to specific clinical parameters, for example, local circumstances, skills, equipment, resources and patient preferences. Adapting the SORs to a local situation is possible if the reason for the choice is sufficiently transparent and this is crucial for successful implementation. Inclusion of patients in clinical trials is an appropriate form of patient management in oncology and is recommended frequently within the SORs, particularly in situations where evidence is too weak to support an intervention.

The type of evidence underlying any ‘*Standard*’, ‘*Option*’ or ‘*Recommendation*’ is indicated using a classification developed by the FNCLCC based on previously published models. The level of evidence depends not only on the type and quality of the studies reviewed, but also on the concordance of the results (Table 2

**Table 2:** Definition of level of evidence

*Level A*
There exists a high-standard meta-analysis or several high-standard randomised clinical trials that give consistent results

*Level B*
There exist good quality evidence from randomised trials (BI) or prospective or retrospective studies (B2). The results are consistent when considered together

*Level C*
The methodology of the available studies is weak or their results are not consistent when considered together

*Level D*
Either the scientific data do not exist or there is only a series of cases

*Expert agreement*
The data do not exist for the method concerned, but the experts are unanimous in their judgement

). When no clear scientific evidence exists, judgement is made according to the professional experience and consensus of the expert group (‘expert agreement’).

The document containing the Standards, Options and Recommendations was then reviewed by a group of independent experts (see Appendix) and after taking into consideration their comments, the guidelines were validated by the working group.

This is a translation of the summarised version based on the full text (in French) which has been published as a monograph (Villers *et al*, 2002) and which is also available on the FNCLCC web site (http://www.fnclcc.fr). These clinical practice guidelines will be updated when new evidence becomes available or if there is a new consensus among the experts.

## RISK FACTORS

Family history (inherited or familial forms) and ethnogeographical origins (particularly African) enable high-risk groups to be defined as a priority for targeted screening (annual digital rectal examination and serum PSA determination in men over 40 years old).

## CLASSIFICATION OF PROSTATE ADENOCARCINOMA

The 1997 TNM classification, modified by the criteria of the *American Joint Cancer Committee* for stage T1a and T1b cancers, should be used (standard). A cancer should be classed as stage T1a when it accounts for at least 5% of the resected tissue (by transurethral or simple prostatectomy) with a Gleason score under 7 (i.e. no grade 4 or 5 disease) (standard).

Prior to surgery, patients should be stratified by their initial PSA concentration, the ratio of number of positive biopsies to the total number of biopsies and the percentage of grade 4/5 disease, in order to evaluate the response to treatment (option). Histopathological findings from the radical prostatectomy specimen should be documented (option), as they are independently correlated with prognosis (i.e. the risk of PSA recurrence after 5 years). The three relevant criteria are the total tumour volume, the extent of any poorly differentiated tumour (Gleason grades 4 and 5) and tumour localisation outside or within the transition zone (option).

## PATHOLOGY

### Histological diagnosis on biopsy specimens

The diagnosis of prostatic adenocarcinoma is generally made using routine haematoxylin and eosin staining and is based on structural and cytological findings (standard). If an isolated area of high-grade prostatic intraepithelial neoplasia (PIN) or atypical or borderline lesions are detected, serial sections of all resected tissue should be made to locate any foci of microscopic carcinoma (standard). Anti-PSA and anti-PAP antibodies can be used to detect metastatic sites in those patients with poorly differentiated tumours of uncertain prostatic or urethral origin (standard).

Immunohistochemistry with anti-cytokeratin 903 antibodies is a complementary diagnostic tool. The results should be interpreted in conjunction with the histological findings obtained using standard techniques (option).

### Gleason score

The Gleason grading system is the standard staging system and the following rules should be applied:

- the Gleason grade(s) apply(ies) to the dominant growth patterns;
- the Gleason score corresponds to the sum of the two dominant grades; when three grades are present, the highest grade and the dominant grade should be used;
- the modified Gleason score should indicate the proportion of grade 4 and 5 disease present (option).

Tumour grade should not be assessed in those patients who have been treated with radiotherapy or hormonal therapy. The use of the modified Gleason score, that is, proportion of grade 4 and 5 disease, is recommended.

### Histological types and histoprognostic factors

The majority of malignant prostatic tumours are adenocarcinomas originating from the glands in the peripheral and transition zones. The histoprognostic factors that should be determined are: histological type; the modified Gleason score and the Gleason score; the pathological classification (TNM 97); extraprostatic extension; invasion of the seminal vesicles; the status of the margins and the nodal status (standards). Two other factors can be taken into consideration: perineural invasion and tumour volume (options).

### Pathology report

#### Prostatic biopsies

The pathology report for prostate biopsies should specify: the length of the biopsy core in millimetres; its quality (mentioning any breaks); the length of tumour involvement in millimetres or as a percentage of the biopsy length; the Gleason score and the presence of any capsular, pericapsular or extraprostatic extension (standard). Only high-grade PIN lesions should be noted. If these are isolated, all biopsy tissue should be examined for the presence of infiltrating microfoci.

The report can contain a diagrammatic representation (or table) of the results and the modified Gleason score (proportion of grades 4 and 5) (options). In the conclusion, the number and localisation of affected areas should be summarised in an agreed format.

In the absence of any definite malignant change, dystrophic lesions described in the report should not be mentioned in the conclusion, unless there is extensive destruction due to prostatitis (recommendation).

#### Transurethral resection specimens

All the resected cores, up to eight blocks, should be included (standard). When there are large amounts of tissue, an extra block should be included for each 5 g of resected tissue (standard). In certain situations, depending on the clinical context (e.g., a young patient or an elevated PSA concentration), it may be necessary to analyse all resected tissues immediately. There is no consensus as to the number of histological blocks that should be made from tissue from a simple suprapubic prostatectomy. A minimum of one block for every 5 g of tissue should be fixed, depending on the macroscopic appearance of the tissue (recommendation).

The report should specify: the histological type of the cancer; the percentage of Gleason grades 4 or 5 and the Gleason score; the proportion of involved cores as a percentage of the total number of cores; and any extraprostatic extension (standard). The report should include (option): presence of perineural invasion; vascular invasion; PIN foci; post-therapeutic changes; atypical adenomatous hyperplasia and benign hypertrophy.

#### Radical prostatectomy specimen

The prostate should be examined using the Stanford serial section technique. The report should specify: histological type; Gleason score; pathological stage (pTNM, 1997); presence of extraprostatic and seminal vesicle invasion and the status of margins (standard). The report can include: the proportion of grades 4 and 5 (modified Gleason score); tumour volume (estimated in terms of the volume of the gland); tumour localisation (peripheral or transitional zone – benign hypertrophy); any associated lesions; perineural invasion; microvascular invasion and post-therapeutic changes (option). The inclusion of all prostatic sections guarantees the best assessment of the excision margins (recommendation).

#### Pelvic lymphadenectomy specimen

A frozen section can be examined prior to the definitive histopathological evaluation (option). The latter, performed following fixation, should include all nodes removed. They should be sectioned at several levels to increase the likelihood of detecting any micrometastases (standard).

## METHODS OF DETECTION (FIGURES 1 AND 2)

### Rectal examination

**Figure 1:**
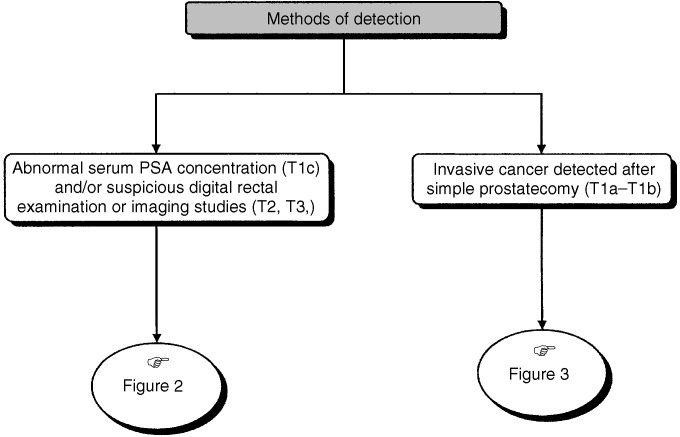
Tumour stage according to method of detection.

**Figure 2:**
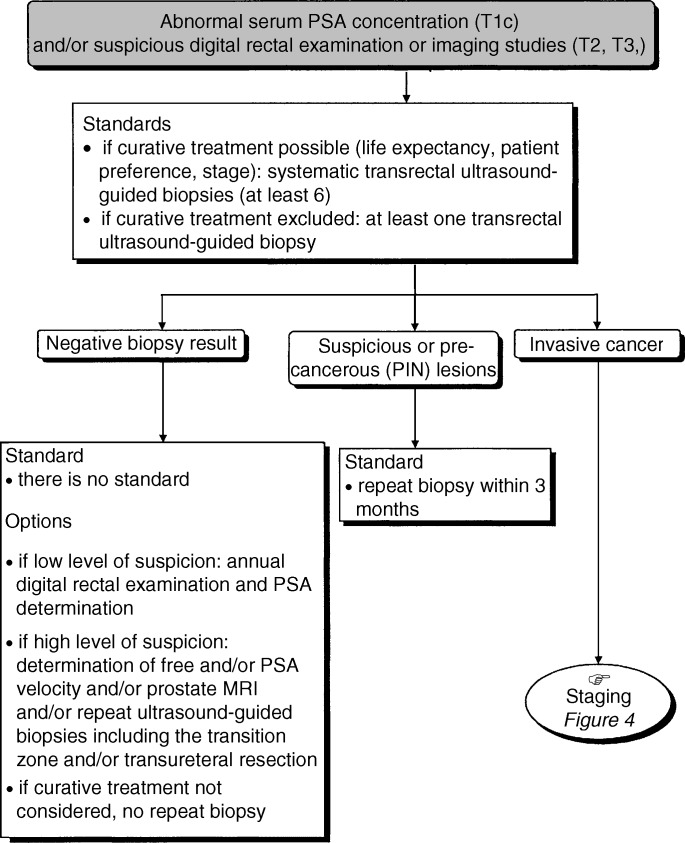
Diagnostic investigation for patients with abnormal serum PSA concentration (T1c) and/or suspicious digital rectal examination or imaging studies (T2, T3,).

Any anomaly detected during digital rectal examination suggestive of prostate cancer in the absence of infection should be investigated further with a transrectal ultrasound-guided biopsy even if the PSA concentration is normal (standard).

### PSA determination

Total serum PSA determination (upper limit of the reference range: 4 *μ*g l^−1^) remains the reference test for screening and the primary indication for biopsy (standard). The value of free PSA determination in cancer screening remains to be determined. There is no consensus as to how often PSA concentrations should be determined. Repeated PSA determinations should be performed by the same laboratory using the same technique (standard). A lower upper limit of the reference range for total serum PSA concentration (between 2 and 4 *μ*g l^−1^) can be used in men under 65 years old or those at risk (option). The adjustment of the upper limits of the reference range for PSA concentration, using age and prostatic volume (PSA density), has not been validated against the decision to perform biopsies (recommendation).

### Imaging

There is no indication for imaging in the primary diagnostic work-up (standard). Magnetic resonance imagery (MRI) and colour Doppler ultrasound are under evaluation for the detection of cancer following a negative result from initial biopsies (recommendation).

### Prostatic biopsy

A diagnosis of prostate cancer is made following the histopathological examination of prostatic biopsy samples (standard). Transurethral resection is not recommended as a first-line biopsy if prostate cancer is suspected (standard). In potentially curative situations, at least six systematic transrectal ultrasound-guided biopsies, sampling particularly the posterior zone, should be taken. The aim and the practical aspects of this investigation should be explained to the patient (standard). Rectal preparation by enema and prophylactic antibiotics effective against Gram negative bacteria should be performed to prevent infectious complications (standard).

The biopsies can be performed either in a day-hospital or outpatient setting, usually with local anaesthesia only (option). In a minority of patients, locoregional or general anaesthesia may be necessary. Additional biopsies may be performed on any zones found to be abnormal on clinical or ultrasound examination (option). When curative treatment is not planned, fewer biopsies can be performed (option). A more extensive procedure with 10 biopsies can be undertaken if the first series of biopsies gives negative results. The patient should be informed about the risks of this investigation. They must have the contact details of the emergency department they should contact, if any complications should occur.

#### Indications and strategies for further biopsies after the diagnosis of PIN or suspicious lesions (Figure 2)

Only high-grade PINs should be noted on the histopathological report of the biopsy (standard, level of evidence: B2). The diagnosis of a high-grade PIN should not lead to a treatment plan (standard, expert agreement). A further series of biopsies should be performed within 3 months in situations where PIN or suspicious lesions have been diagnosed (standard, expert agreement). When curative treatment is not planned (life expectancy of less than 10 years, patient's choice, etc.), further biopsies are not recommended (recommendation).

#### Further prostate biopsies after initial negative biopsy (Figure 2)

When curative treatment is not planned, an additional work-up is not indicated (standard). When curative treatment is planned there are two possible options: (1) no further biopsy; (2) wait for 3 months and then re-evaluate with serum PSA determination and ultrasound-guided biopsy. Depending on the degree of suspicion, the additional work-up for re-evaluation can include: PSA velocity and the percentage of free PSA; a further series of biopsies including the transition zone (the number of biopsies can be increased by including laterally directed biopsies in the peripheral zone) or a transurethral resection (option). If the test result is suspicious (PSA concentration, digital rectal examination), a second series of biopsies is recommended (recommendation).

#### Management of patients following a diagnosis of stage T1a or T1b cancer (Figure 3)

**Figure 3:**
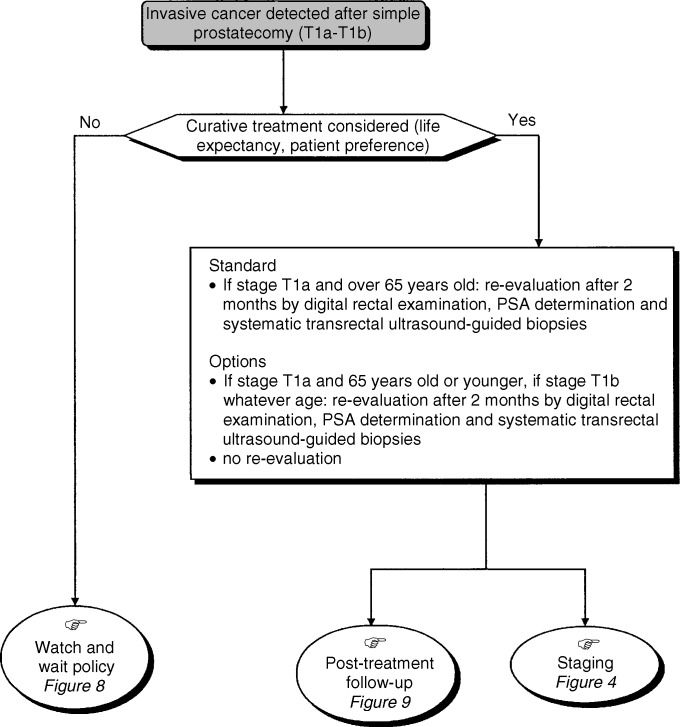
Management of patients with stage T1a and T1b tumours.

There are two therapeutic options possible for patients with a life expectancy of more than 10 years: primary curative treatment or evaluation of the remaining prostate to confirm the presence of residual tumour. The remaining prostate should be biopsied if the postoperative digital rectal examination is abnormal, and/or the 3-month postoperative PSA concentration is higher than 4 *μ*g l^−1^ or has been reduced by less than 50%. If the results of the biopsies of the remaining prostate are negative, clinical surveillance and determination of serum PSA concentrations should be performed every 6 months (option). If the biopsies of the remaining prostate are positive, curative treatment should be undertaken. Curative treatment is not recommended for patients with a life expectancy of less than 10 years (recommendation, expert agreement).

## STAGING (FIGURE 4)

### Staging by clinical examination and imaging

**Figure 4:**
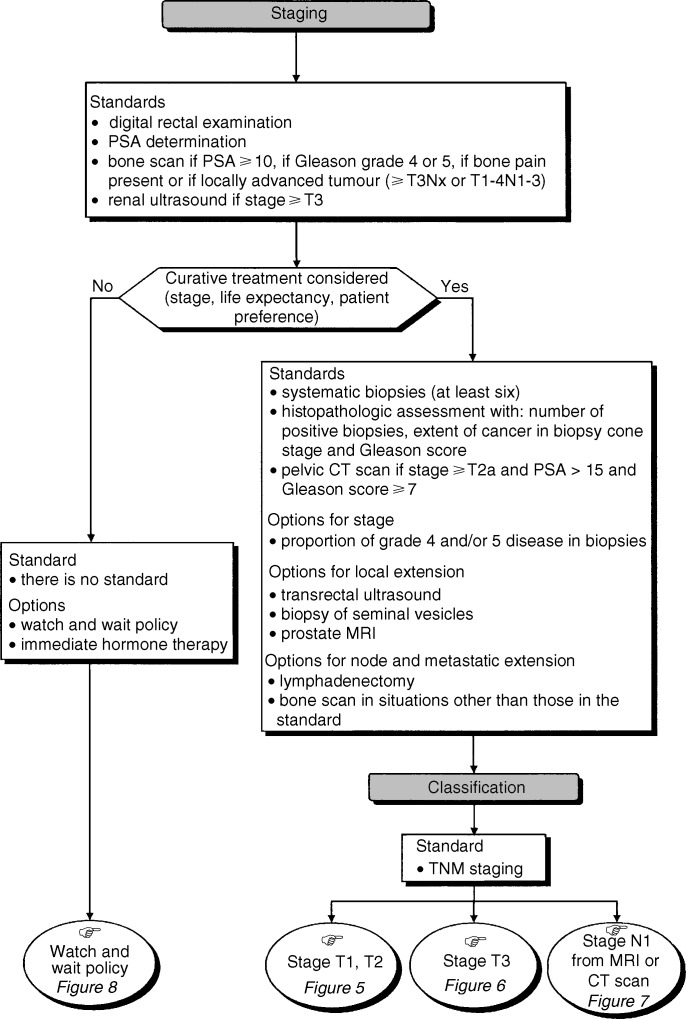
Staging for a histologically proven invasive cancer.

Digital rectal examination and a transrectal ultrasound should be performed prior to, and used as a guide for, biopsies of the periprostatic tissue and seminal vesicles (standard). A renal ultrasound and CT scan should be performed for patients with stage T3 cancer (standard). Pelvic or endorectal coil MRI can be performed if radical prostatectomy or radiotherapy is indicated, if extraprostatic extension is suspected and if the results could modify the treatment plan (option).

### Evaluation of periprostatic metastatic spread

Periprostatic metastatic spread can be evaluated by biopsy of the seminal vesicles or the periprostatic tissue (option). These biopsies should be taken at the same time as the first series of prostatic biopsies if the results of the digital rectal examination, imaging or the PSA concentration are suggestive of periprostatic involvement (recommendation). Biopsies of the seminal vesicles should be taken, as a second-line investigation, if the results of the biopsies of the bases of the two prostatic lobes are positive (recommendation).

### Imaging for node involvement

Abdominal and pelvic CT scan should be performed in patients with: T2a stage disease or higher, a PSA concentration greater than 15 *μ*g l^−1^ and a Gleason score of at least 7 (standard). MRI for the same indications is optional.

### Lymphadenectomy for staging

Lymphadenectomy should be limited to the ilio-obturator regions (standard) and should be performed in those patients undergoing radical prostatectomy, (standard). It may not be necessary to perform a lymphadenectomy at the same time as radical prostatectomy if the patient has good prognostic factors: that is, stage T1 tumour; a Gleason score of under 6 and a pretherapeutic PSA concentration of less than 10 *μ*g l^−1^ (option). An isolated lymphadenectomy should not be undertaken prior to radiotherapy. It can be performed if the risk of node invasion is high (option).

### Assessment of bone metastases by bone scan

Irrespective of the planned treatment, a bone scan is indicated during the initial work-up in the presence of one of the following: bone pain; a locally advanced prostatic lesion (at least T3Nx or T1-4N1-3 or higher); the presence of Gleason grade 4 or 5 and a PSA concentration of at least 10 *μ*g l^−1^ (standard). The interpretation of the bone scan should be made in light of the patient's PSA concentration and clinical history (recommendation). In situations other than those defined in this standard, the decision to undertake a bone scan is left to the discretion of the physician, with the knowledge that the average risk of bone metastases in this situation in Europe is 3.3% (option, expert agreement). Additional studies are needed to assess the prevalence of bone metastases in situations other than those defined in the standard (recommendation).

## FACTORS TO CONSIDER WHEN MAKING A TREATMENT PLAN (FIGURES 5, 6 and 7)

### Criteria for assessing response to curative treatment

**Figure 5:**
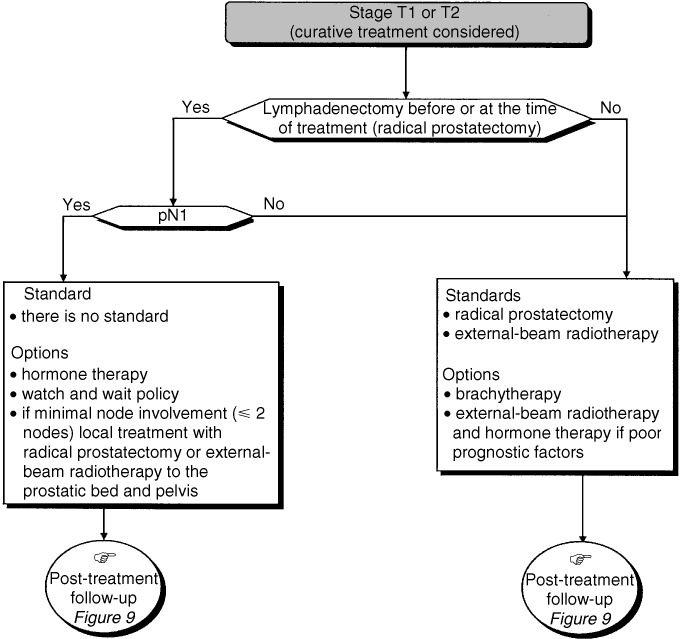
Therapeutic management of patients with stage T1 or T2 tumours.

**Figure 6:**
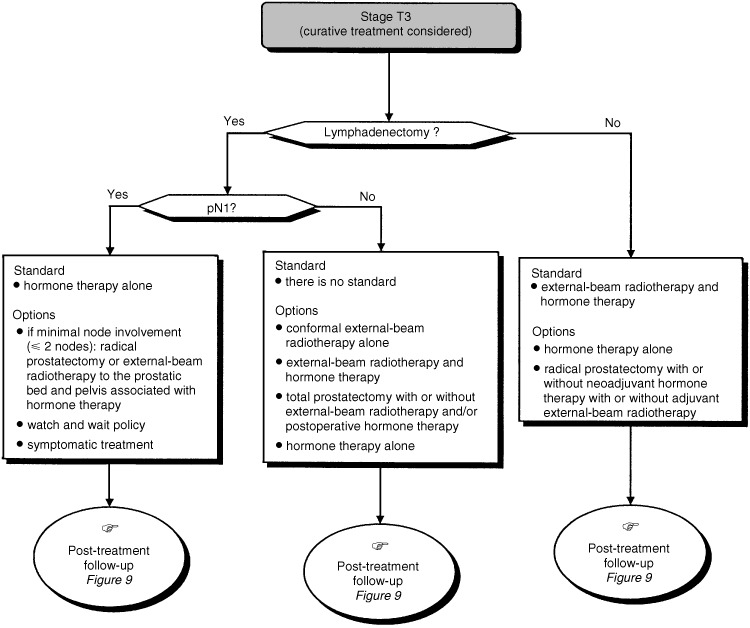
Therapeutic management of patients with stage T3 tumours.

**Figure 7:**
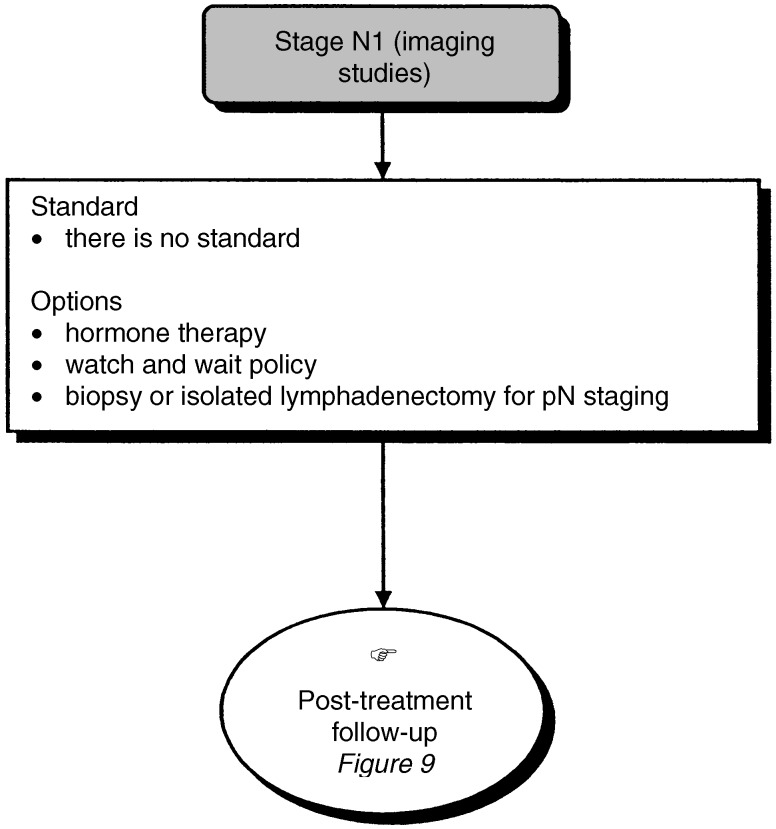
Therapeutic management of patients with stage N1 tumour (imaging studies).

Screening using PSA concentration can detect stage T1c disease and enables the detection of prostate cancer 4–5 years earlier than the stage T2 tumours reported in early series. The median actuarial delay between the appearance of metastases and death is 5 years. Specific follow-up for a minimum of 15 years is necessary to evaluate the efficacy of treatment of localised prostate cancer (standard). A 10-year metastasis-free survival is an acceptable criteria for evaluating treatment response for localised prostate cancer (standard). The criterion for complete remission after radical prostatectomy is an undetectable PSA concentration (under 0.1 *μ*g l^−1^) for at least 7 years after radical prostatectomy (standard). The criteria for complete remission after external-beam radiotherapy or brachytherapy have not yet been defined (standard). The criterion for progression after radical prostatectomy, external-beam radiotherapy or brachytherapy is an increase in PSA concentration measured on three successive occasions at monthly intervals (standard). The median delay between an increased PSA concentration and the appearance of metastases is 8 years (standard).

### Prognostic factors related to the prostate tumour

Clinical tumour stage, Gleason score and the pretreatment PSA concentration are prognostic factors for locoregional metastatic spread and therefore, for treatment response (standard). Other prognostic factors that can be used are: the Gleason grades present; the number of affected biopsies; the extent of the tumour tissue in the core biopsy and perineural invasion (option). Partin tables can be used, before treatment, to evaluate the risk of extraprostatic metastatic spread and pelvic node invasion (option).

### Patient-related prognostic factors

Curative treatment should be offered to men with localised prostate cancer if their life expectancy is at least 10 years. Life expectancy can be estimated using life tables for the general population and the presence or absence of comorbidities likely to have an impact on mortality.

## TREATMENT: WATCH AND WAIT POLICY (FIGURE 8)

**Figure 8:**
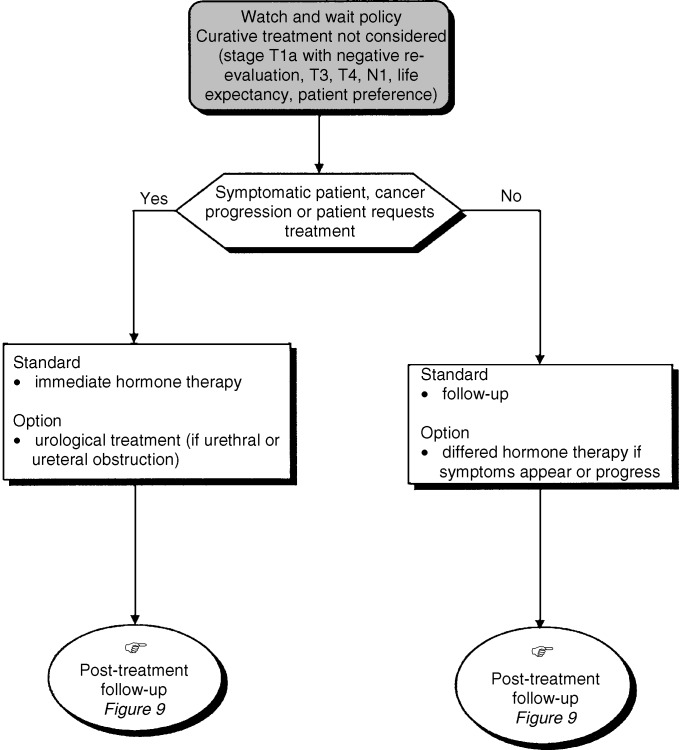
Watch and wait policy.

For patients with stage T1c or T2 prostate cancer with a life expectancy of less than 10 years, a watch and wait policy can be considered (option, level of evidence: C). In patients with a life expectancy of less than 10 years, a watch and wait policy is even more appropriate for lower stage disease and grade (recommendation).

## TREATMENT: RADICAL PROSTATECTOMY

Prostatectomy is an effective treatment for stage T1a, T1b, T1c or T2 prostate cancer (standard, level of evidence: B2). Prostatectomy can be considered for stage T3 and pN1 cancers (option). Prostatectomy should be undertaken in stage T3 and pN1 cancers only in the setting of a randomised clinical trial assessing the efficacy of prostatectomy alone or in combination with other treatment (radiotherapy or hormone therapy) (recommendation). Radical prostatectomy is not recommended for stage pN1 high-grade tumours (Gleason score >7) (recommendation).

## TREATMENT: EXTERNAL-BEAM RADIOTHERAPY

External-beam radiotherapy alone is an effective treatment for stage T1, T2 or T3, N0, M0 prostate cancer (standard, level of evidence: B2). The minimum recommended dose is 70 Gy, irrespective of the prognostic factors present (recommendation, level of evidence: B2). Patients should be included in randomised clinical trials assessing dose escalation.

### Quality control for external-beam radiotherapy

Dosimetry, with CT planning, is recommended for defining the target tumour volume (recommendation, level of evidence: B2).

### Toxicity of external-beam radiotherapy

Two types of external-beam radiotherapies are possible: conformal or conventional (option, level of evidence: C). Conformal radiotherapy reduces late toxicity compared with conventional radiotherapy and should be used when giving high doses (recommendation, level of evidence: C). Factors reported to affect the risk of complications following external-beam radiotherapy are transurethral prostatic resections prior to radiotherapy and the dose of external-beam radiotherapy used.

### Dose escalation

Patients with a good prognosis (T1–T2a, PSA<10 *μ*g l^−1^ and Gleason score between 2 and 6) have not been shown to benefit from dose escalation above 70–74 Gy. Patients with an intermediate prognosis receiving radiotherapy alone seem to benefit most from a dose escalation above 74 Gy.

## TREATMENT: BRACHYTHERAPY

Retropubic brachytherapy for prostate cancer should no longer be used (standard, level of evidence: C).

### Brachytherapy with temporary implants

#### Technical data

The isotope that should be used for brachytherapy is Iridium^192^ (standard). Two types of brachytherapy can be used: low-dose rate or high-dose rate (option).

#### Indications

Dosimetric planning should be used. The combination of brachytherapy with temporary implants and external-beam radiotherapy is a therapeutic option for locally advanced prostatic cancer (option, level of evidence: D). Brachytherapy with temporary implants should not be used for stage T1 or T2a tumours outside the setting of a randomised clinical trial (recommendation).

### Brachytherapy with permanent implants

#### Technical data

Dosimetric planning should be used either prior to implantation or during the procedure (standard). The recommendation from Task Group 43 (TG-43) is that brachytherapy dosimetric parameters should be used for calculating the dose (standard, expert agreement). Postimplantation dosimetry should be performed 4 weeks and 2–3 weeks after the implantation for iodine 125 and palladium 103, respectively (standard). The patient should be given information about radio-protective measures for children and pregnant women, the need to use condoms and to filter all urine and the need to inform physicians in the event of a pelvic intervention (standard, expert agreement). Training in brachytherapy techniques is essential and should be evaluated (standard, level of evidence: B2).

It is not known whether freehand implantation or US/CT-guided template systems give better results (option, level of evidence: D). Similarly, there is no evidence that iodine 125 should be used in preference to palladium 103 (option, level of evidence: C).

Transrectal ultrasound data should be used for predictive dosimetry and implantation (recommendation, expert agreement). The modified peripheral implantation technique is recommended to minimise the risk of urethral overdose (more than 200% of the prescribed dose) (recommendation, expert agreement). The minimum peripheral doses recommended are 144 Gy for iodine 125 in monotherapy and 100–110 Gy in combination with radiotherapy (40–50 Gy) and 115 and 80–90 Gy for palladium 103 (recommendation, expert agreement). The report should specify: the volume implanted; the number of seeds implanted; the number of needles used; the total activity and the prescribed dose. Dosimetry should be performed following implantation using CT scanning, with the calculation of dose-volume histograms (DVHs). The recommended delay for postimplantation dosimetry is 4 weeks for iodine 125 (and 2–3 weeks for palladium 103). The data to be reported are:

- D100, D90 and D80: isodose covering 100 (minimal peripheral dose), 90 and 80%, respectively, of the prostatic volume. A good-quality implant will have at least a D90 of the dose prescribed;
- V200, V150, V100, V90 and V80: the percentage of the prostatic volume receiving 200, 150, 100, 90 and 80%, respectively, of the dose prescribed;
- the total volume of the prostate obtained for the postimplantation dosimetry;
- the delay between the brachytherapy and the postimplantation dosimetry;
- the doses received by the ureter and the anterior wall of the rectum.

#### Indications

Prostatic brachytherapy alone using permanent implants is potentially curative in patients with the following characteristics: clinical stage T1 or T2a disease (TNM 1992), a Gleason score of 6 or lower and a PSA concentration of less than 10 *μ*g l^−1^ (option). Continued evaluation of this treatment is recommended (morbidity, tumour control) in the setting of a randomised clinical trial (recommendation).

#### Brachytherapy toxicity

Brachytherapy is contraindicated in patients who have undergone a previous large transurethral resection (standard) and in patients with a previous limited transurethral resection (recommendation). A prostatic volume of more than 50–60 cm^3^ and/or the presence of hypertrophy in the median lobe are relative contra-indications for brachytherapy (recommendations). The modified peripheral implantation technique is recommended.

The use of questionnaires to evaluate urinary function prior to implantation is recommended (level of evidence: C). The length of ureter receiving more than 200% of the prescribed dose should be reported. The maximum length of the rectum receiving 100 and 120% of the prescribed dose should be limited to 10 and 5 mm, respectively (expert agreement).

### Prostatic biopsy to measure treatment response to brachytherapy

Biopsies should not be performed until 18–24 months after brachytherapy (recommendation).

## TREATMENT: HORMONAL THERAPY ALONE

Hormone therapy alone for stage T1, T2, Nx or M0 cancer is not indicated in the absence of progressive disease (standard). Hormone therapy, either alone or in combination, can be considered in patients with nonmetastatic disease if curative treatment is not planned (option). This treatment modality is under evaluation.

## TREATMENT: CHEMOTHERAPY

The use of chemotherapy in nonmetastatic prostate cancer is not recommended (option).

## COMBINED TREATMENTS

### Brachytherapy and hormonal therapy

The combination of brachytherapy and hormonal therapy may be beneficial, compared with brachytherapy alone, in patients with an intermediate prognosis (Gleason score of more than 7 and/or PSA concentration higher than 10 *μ*g l^−1^) (option, level of evidence: C). Combined brachytherapy and hormonal therapy should only be proposed in the setting of a randomised clinical trial (recommendation).

### Brachytherapy and external-beam radiotherapy

The combination of external-beam radiotherapy and brachytherapy with permanent implantation can be considered in patients with an intermediate prognosis (option). The benefits of this combination should be evaluated against external-beam radiotherapy alone, or in combination with hormonal therapy in the setting of a randomised clinical trial (recommendation).

### Brachytherapy, external-beam radiotherapy and hormonal therapy

The combination of brachytherapy, external-beam radiotherapy and hormonal therapy for patients with a poor prognosis should only be considered in the setting of a randomised clinical trial (recommendation).

### Neoadjuvant hormonal therapy before prostatectomy

There is no benefit from neoadjuvant hormonal therapy for local stage cancers (T1–T2) (standard, level of evidence: B1). Neoadjuvant hormonal therapy before radical prostatectomy is not indicated (option). Neoadjuvant hormonal therapy should not be considered for stage T3 cancers, outside the setting of a randomised clinical trial (recommendation, level of evidence: B1).

### Prostatectomy and adjuvant hormonal therapy

Adjuvant hormonal therapy can be prescribed after radical prostatectomy for patients with node involvement (option, level of evidence: C). Patients with stage pT3 cancer and positive margins should be included in randomised clinical trials to determine the efficacy of adjuvant hormonal therapy (recommendation).

### Prostatectomy and adjuvant radiotherapy

In patients with node involvement (pN1), adjuvant radiotherapy after radical prostatectomy has not been shown to improve outcome (option, expert agreement). Adjuvant radiotherapy may be considered in patients with widespread stage pT3a cancer, or with stage pT4 disease without node or seminal vesicle invasion or with positive surgical margins, particularly if the postoperative PSA concentration cannot be determined (option, level of evidence: C). One potential advantage of adjuvant radiotherapy is that the dose is lower than that used when treatment is deferred until a rise in PSA is detected. Patients should be included in randomised clinical trials to determine the efficacy of adjuvant radiotherapy.

### Hormonal therapy and radiotherapy

The combination of radiotherapy and long-term hormone therapy can be considered in patients with locally advanced prostatic cancer, stages T2b, T3, and/or Gleason score of at least 8 (option). Short-term hormone therapy can be prescribed for patients with a good prognosis (option). Since the optimal combination of hormone and radiotherapy has not been established, combination treatment should be used only in the setting of a randomised clinical trial (recommendation).

### Treatments under evaluation

Neutron therapy and transrectal treatment with targeted high-intensity ultrasound are under evaluation. The efficacy of these treatments should be assessed against the newest radiotherapy techniques (recommendation).

## POST-TREATMENT FOLLOW-UP (FIGURE 9

### Follow-up after prostatectomy

**Figure 9:**
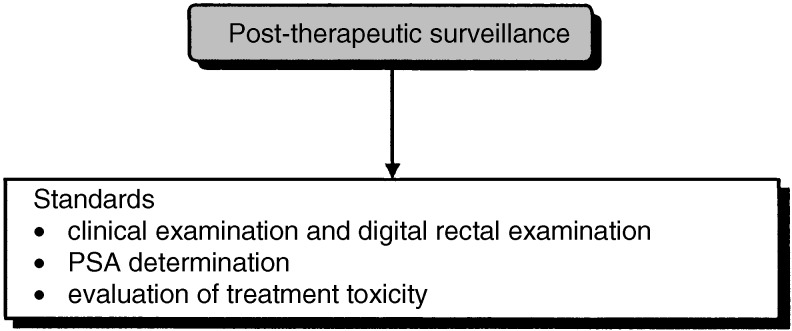
Post-treatment follow-up.

Total serum PSA should be measured between 1 and 3 months after radical prostatectomy (standard). Total serum PSA levels should be measured every 3 months during the first year (or less frequently if the concentration is below the limit of detection) and every 6 months for the following 7 years if the concentration is below the limit of detection (standard, level of evidence: B2). Digital rectal examination is optional in patients with a total serum PSA level below the limit of detection (option, expert agreement).

### Follow-up after radiotherapy

After radiotherapy, follow-up should include PSA determination and digital rectal examination for an indefinite period (standard, expert agreement). PSA determination and digital rectal examination should be undertaken every 6 months (recommendation, expert agreement). Prostatic biopsies are not necessary if the total serum PSA concentration is low (recommendation).

## Appendix

### Reviewers

A Alberti-Mourret (Centre Hospitalier, Belfort), P Arveux (Hôpital Saint-Jacques, Besançon), M Assicot (Institut Gustave Roussy, Villejuif), JC Baron (Clinique Saint-Grégoire, Tours), V Beckendorf (Centre Alexis Vautrin, Vandoeuvre-lès-Nancy), Ph Beuzeboc (Institut Curie, Paris), P Bey (Centre Alexis Vautrin, Vandoeuvre-lès-Nancy), L Boccon-Gibod (CHU Hôpital Bichat-Claude Bernard, Paris), A Boneu (Institut Claudius Régaud, Toulouse), JL Bonnal (Hôpital Victor Provo, Roubaix), B Bouabana (Vizille), M Bouc (Nantes), C Carpuat (Bagnères-de-Bigorre), M Caudry (Hôpital Saint Andrè, Bordeaux), L Chauveinc (Institut Curie, Paris), B Chauvet (Clinique Sainte Catherine, Avignon), F Cornud (avenue Robert Schuman, Paris), JM Cosset (Institut Curie, Paris), A Couanon (Saint-Cloud), C Coulange (CHU Hôpital Salvator, Marseille), B Cuzin (Lyon), JL Davin (Clinique Rhône-Durance, Avignon), C Dejax (Centre Jean Perrin, Clermont-Ferrand), A Delage (Saint-Michel), G de Pinieux (Groupe hospitalier Cochin, Paris), G Ph Desbonnets (Fleurbaix), JF Douard (Clinique Saint-Dominique, Flers), JB Dubois (Centre Val d'Aurelle, Montpellier), B Dubray (Centre Henri Becquerel, Rouen), I Durand-Zaleski (Hôpital Henri Mondor, Créteil), G Escourrou (CHU Rangueil, Toulouse), R Fourcade (CH d'Auxerre, Auxerre), Y Fulla (Hôpital Cochin, Paris), JP Gérard (Centre Hospitalier Lyon-Sud, Pierre-Bénite), F Giammarile (Centre Léon Bérard, Lyon), Ph Gomez (Rouen), R Gonthier (CHU Hôpital de la Charité, Saint-Etienne), J Goussard (Centre François Baclesse, Caen), F Guerber (SCM B 12, Grenoble), B Guilloneau (Institut Mutualiste Monsouris, Paris), L Guy (Hôpital Gabriel Montpied, Clermont-Ferrand), P Hardouin (Institut Calot, Berck), O Hélénon (Hôpital Necker, Paris), A Houlgatte (HIA du Val de Grâce, Paris), F Iborra (Montpellier), J Irani (CHU La Milétrie, Poitiers), G Kouri (boulevard de Vésone, Périgueux), J Lambert (Saint-Avold), E Lartigau (Centre Oscar Lambret, Lille), S Legrain (Hôpital Bichat, Paris), L Lemaître (CHRU Hôpital Huriez, Lille), D Léonard (Boulogne-sur-Mer), E Leprise-Fleury (Centre Eugène Marquis, Rennes), A Lesourd (IMM Choisy, Paris), JP Maire (Hôpital Saint André, Bordeaux), C Mazerolle (CHU Purpan, Toulouse), JJ Mazeron (CHU la Salpétrière, Paris), JF Minne (Clinique radiologique des Dentellières, Valenciennes), B Minsky-Kravetz (Luce), JL Moreau (Nancy), N Mottet-Auselo (CHU G Doumergue, Nîmes), TD Nguyen (Institut Jean Godinot, Reims), Y Otmezguine (Hôpital Henri Mondor, Créteil), P Paycha (Clinique Escudié, Albi), M Petit (Hôpital Robert Debré, Paris), S Piperno-Neumann (Hôpital Avicenne, Bobigny), I Resche (Centre René Gauducheau, Saint-Herblain), JP Rieu (Centre hospitalier, Albi), F Rocher (Clinique Sainte Marie, Chalon-sur-Saône), F Rousselot (Clinique Saint-Germain, Brive), A Ruffion (rue Bataille, Lyon), H Sancho-Garnier (Centre Val d'Aurelle, Montpellier), JB Sautron (Bagnols-en-Forêt), P Schaffer (Laboratoire d'épidémiologie et de santé publique, Strasbourg), JM Simon (Hopital Pitié-Salpétrière, Paris), JY Soret (CHU Clinique Urologique, Angers), G Souweine (Conseil scientifique de l'Andem, Venissieux), F Staerman (CHU de Reims, Reims), M Steinling (CHRU Hôpital Huriez, Lille), A Vieillefond (Groupe hospitalier Cochin, Paris) and JJ Voigt (Institut Claudius Régaud, Toulouse).

This practice guideline has been developed with the collaboration of the Association Française d'Urologie (AFU).
